# Clinical outcomes and feasibility of a fast-track protocol after total ankle replacement: a prospective study

**DOI:** 10.1007/s00402-026-06532-9

**Published:** 2026-10-20

**Authors:** Luca Maria Vena, Lucrezia Moggio, Erminia Cofano, Umile Giuseppe Longo, Pim van Dijk, C. Niek van Dijk, Giorgio Gasparini, Michele Mercurio

**Affiliations:** 1 Scarnati Clinic, Cosenza, Italy; 2 Rehabilitation Unit, Ospedale degli Infermi, Biella, Italy; 3 Department of Orthopaedic and Trauma Surgery, Magna Graecia University, Catanzaro, Italy; 4 Fondazione Policlinico Universitario Campus Bio-Medico, Roma, Italy; 5 Department of Orthopedic Surgery and Sports Medicine, Amsterdam UMC, Amsterdam Movement Sciences, University of Amsterdam, Amsterdam, Netherlands; 6 Professor Em. University of Amsterdam, Department of Orthopedic Surgery, University of Amsterdam, Amsterdam, Netherlands

**Keywords:** Ankle osteoarthritis, Total ankle replacement, Fast-track protocol, Functional recovery

## Abstract

**Purpose:**

Postoperative recovery after total ankle replacement remains challenging, and evidence supporting the use of fast-track protocols in this setting is limited. The purpose of this study was to evaluate the short-term clinical outcomes and safety of a fast-track protocol following total ankle replacement.

**Methods:**

A prospective observational study was conducted on consecutive adult patients undergoing primary total ankle replacement through an anterior approach between January 2023 and June 2024. All procedures were performed by a single experienced surgeon using the same implant. The fast-track protocol included early postoperative mobilization, multimodal pain management, bleeding control, and structured physiotherapy with standardized discharge criteria. Ankle function was assessed using the American Orthopaedic Foot and Ankle Society’s (AOFAS) Ankle Hindfoot Scale preoperatively and at hospital discharge. Secondary outcomes included time to active ankle mobilization, time to assisted ambulation with crutches, length of hospital stay, and wound healing evaluated using the ASEPSIS wound score.

**Results:**

Twenty-two patients were included. Median AOFAS Ankle Hindfoot Scale scores improved significantly from 29.0 preoperatively to 68.0 at discharge. Active ankle mobilization was achieved after a median of two days, assisted ambulation after three days, and the median hospital stay was five days. Wound healing outcomes were favourable, with low ASEPSIS scores and only one minor wound complication managed conservatively. No major wound complications, infections, or prosthesis-related adverse events were observed.

**Conclusion:**

In this prospective single-arm study, a fast-track protocol following total ankle replacement was feasible and was associated with early functional recovery and favourable short-term safety outcomes. These findings should be interpreted as preliminary and descriptive, given the absence of a control group. Larger comparative studies with longer follow-up are needed to determine its effectiveness relative to conventional postoperative care.

**Supplementary Information:**

The online version contains supplementary material available at https://doi.org/10.1007/s00402-026-06532-9.

## Introduction

Ankle osteoarthritis (OA) is a degenerative condition that impacts quality of life, and its most common aetiology is post-traumatic. Total ankle replacement (TAR) is increasingly identified as a feasible surgical option for patients with end-stage ankle OA, rheumatoid arthritis, or post-traumatic OA [1]. Until recent years, ankle arthrodesis (AA) was considered the gold standard surgical technique for the management of deformity and pain secondary to ankle OA. However, the changes in gait mechanics could potentially result in premature OA of adjacent joints. Unlike AA, TAR preserves joint motion, improves gait biomechanics, reduces theoretically the chance of adjacent joint degeneration, and can potentially enhance overall quality of life [2]. Over the last two decades, innovations in implant design, surgical techniques, and perioperative care have significantly improved outcomes and prosthesis survival [1, 3]. In particular, the evolution from early-generation implants to contemporary fourth-generation TAR designs has resulted in improved primary stability and encouraging short- to medium-term survivorship, ranging from 92% to 98% at 1–2 years for several contemporary systems [4]. More recently, evidence from randomized controlled trials has further supported the clinical effectiveness of TAR compared with ankle arthrodesis, with the TARVA trial demonstrating favourable patient-reported outcomes following TAR at 52 weeks [5]. These advances in implant stability and reliability provide a rationale for reconsidering traditional postoperative strategies and investigating whether earlier mobilization can be safely incorporated into fast-track pathways following TAR. Indeed despite these developments, postoperative recovery following TAR is still challenging. Prolonged immobilisation and delayed mobilization may increase the risk of nosocomial complications, delay the return to daily activities, and contribute to higher healthcare costs [6].Traditionally, a period of postoperative immobilisation and protection of the ankle has been recommended following TAR to allow adequate soft-tissue healing and osseous integration of the implant. In particular, healing of the extensor retinaculum and the surgical wound is an important consideration, while sufficient time for osseous integration is required to achieve stable long-term fixation of the prosthetic components. Insufficient osseointegration may contribute to component loosening or migration.Enhanced recovery or fast-track protocols have been widely adopted in hip and knee arthroplasty, demonstrating consistent benefits in terms of early mobilization, reduced length of stay, lower perioperative complications, and improved patient satisfaction [7–9]. These protocols typically integrate multimodal pain management, early physiotherapy, structured discharge criteria, and patient education [8, 10–13]. However, their application to TAR presents specific challenges. Unlike hip and knee arthroplasty, the ankle is characterized by a relatively thin soft-tissue envelope and limited soft-tissue coverage, particularly over the anterior aspect of the joint. Furthermore, the commonly used anterior surgical approach places the incision at particular risk of wound-healing complications. These anatomical and surgical considerations may explain the traditional emphasis on postoperative immobilisation and protection following TAR and raise concerns regarding the safety of accelerated recovery pathways. In contrast to hip and knee arthroplasty, the application of fast-track protocols in TAR remains limited and poorly studied. Few prospective studies have evaluated early functional recovery, mobilization timelines, and wound-healing outcomes in patients undergoing TAR [10, 14, 15]. Given the rising prevalence of TAR procedures and the increasing demand for high-quality perioperative care, there is a clear need to investigate the feasibility, safety, and potential benefits of fast-track protocols in TAR.

This prospective study aimed to evaluate the short-term clinical outcomes of a fast-track protocol following TAR. We hypothesized that the fast-track protocol would be safe, promote early recovery, and result in improved short-term functional outcomes without increasing wound-related complications.

## Materials and methods

### Study design and setting

This prospective study followed the guidelines defined by the Strengthening the Reporting of Observational Studies in Epidemiology (STROBE) guidelines and checklist.

### Participants

This study included patients who underwent TAR with an anterior approach between January 2023 and June 2024. The inclusion criteria were: (1) age ≥ 18 years, (2) post-traumatic ankle OA or rheumatoid arthritis, (3) availability of complete records, (4) minimum follow-up of 12 months for the assessment of postoperative safety and complications. Patients were enrolled consecutively. Exclusion criteria were: (1) revision of TAR; (2) inability to follow the postoperative rehabilitation protocol; (3) neurological disorder of the lower extremities; and (4) failure to understand or complete the questionnaires.

### Surgical technique

**Fig. 1:**
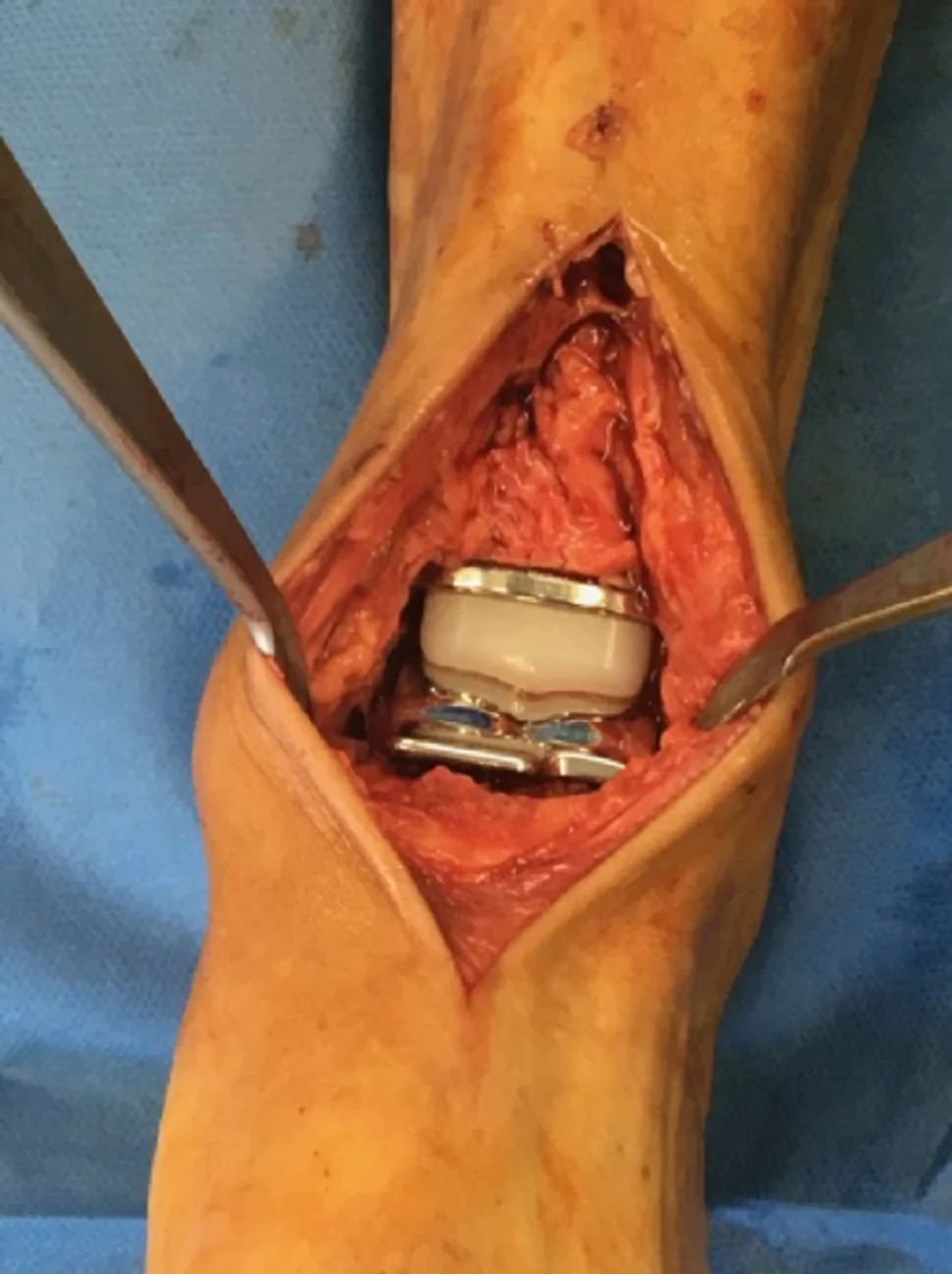
Intraoperative view of a total ankle replacement following anterior approach, showing the implanted prosthetic components in situ

**Fig. 2:**
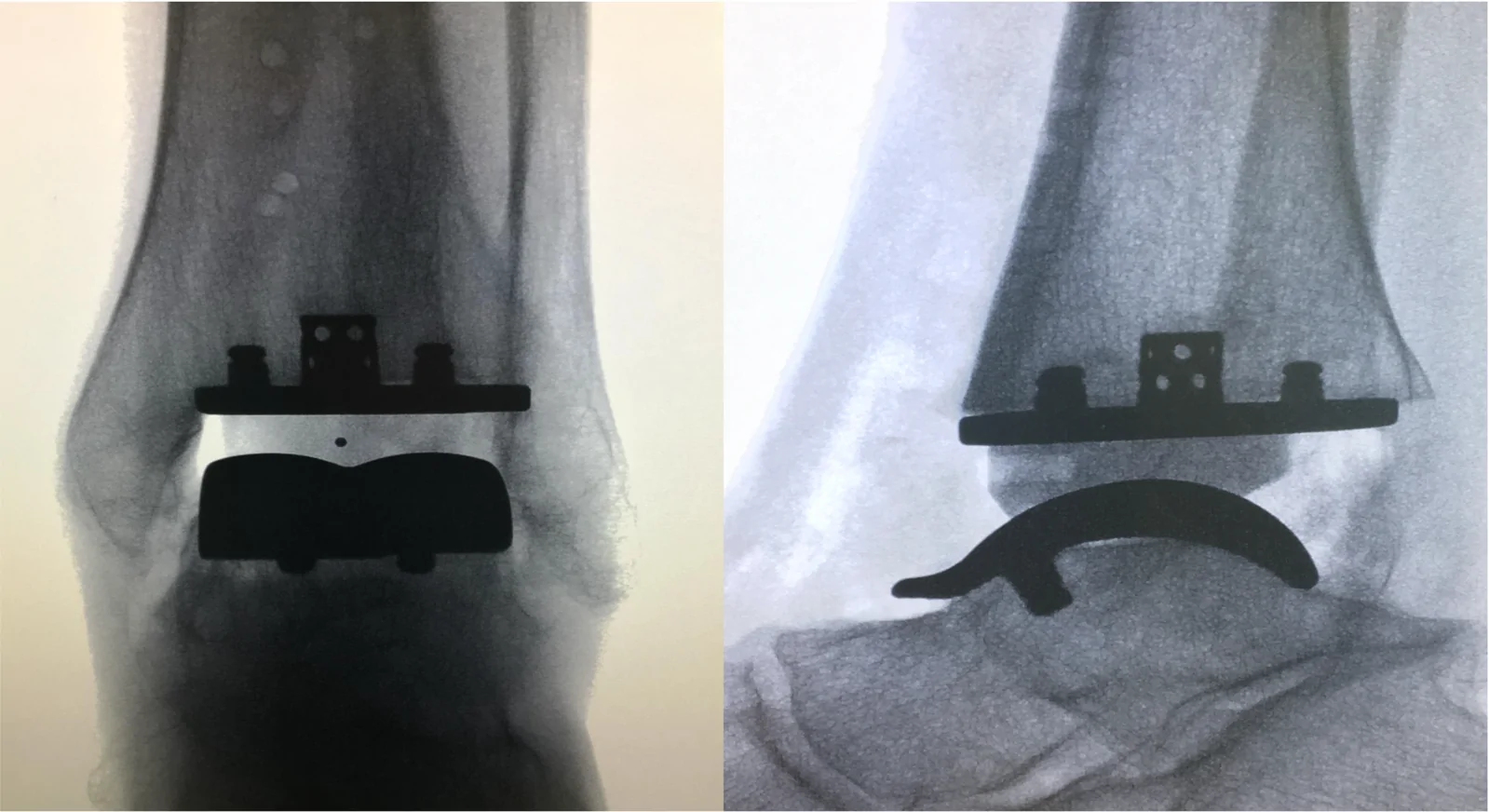
Postoperative anteroposterior and lateral radiographs of the ankle showing a total ankle replacement with well-aligned tibial and talar components

All patients underwent TAR, performed by a single experienced orthopedic surgeon. The surgery was performed under spinal anaesthesia using an anterior approach technique, with the patient in the supine position, and with a pneumatic tourniquet at the thigh level in the absence of specific contraindications. The surgical anterior approach involves the following landmarks: the medial and lateral malleoli and the tibiotalar joint line. A 10–14 cm anterior longitudinal incision was made between the anterior tibial and extensor hallucis longus tendons to expose the extensor retinaculum. When encountered over the retinaculum, the superficial peroneal nerve was identified and protected. Particular care was taken to identify and preserve the anterior tibial neurovascular bundle, consisting of the anterior tibial vessels and deep peroneal nerve, which courses between the extensor hallucis longus and extensor digitorum longus tendons. Once the ankle joint was exposed, capsulotomy and capsulectomy were performed. Osteophytes were removed from the tibia and the talar neck. Tibial and talar cuts were performed under fluoroscopic guidance with a specific tibial guide. The centre of rotation was also located under fluoroscopy, and the tibial and talar component sizes were established. Careful technique was employed to avoid malleolar fractures. The posterior capsule was carefully debrided, and any ossifications were removed. After insertion of the metallic trial components, implant stability and soft-tissue tension were assessed. Finally, the definitive components were inserted, and the wound was carefully closed with an intradermal suture and cyanoacrylate octyl ester skin adhesive (Dermabond, Ethicon Inc., Somerville, NJ, USA). All TARs were performed using the same implant (Vantage Total Ankle System, Advita Ortho, Gainesville, Florida, USA). The range of motion in dorsiflexion and plantar flexion was assessed to ensure that no overstuffing had occurred [16] (Figs. 1 and 2).

### Postoperative fast-track protocol

Postoperatively, the surgical wound was protected with a sterile adhesive dressing and the ankle was immobilized in a walker boot. No specific compressive or silver-containing dressing was used. No additional surgical or wound-related procedures were required during the follow-up period. After the surgery, the subjects followed a fast-track protocol. This management included early postoperative mobilization and cryotherapy at three hours postoperatively. Multimodal pain management was used to facilitate functional recovery, acetaminophen (1 g every 12 h for 5 days), and an elastomeric pump infusion with tramadol 0.1 mg/kg/h for 48 h. Also, an anti-inflammatory therapy with intravenous dexamethasone 16 mg was administered to the patients, 30 min before surgery and 24 h after surgery. Control of bleeding is critical to promote better patient recovery; in this case, the following were administered: intravenous 2 g of tranexamic acid 20 min before surgery, intra-articular 1 g of tranexamic acid when the capsule was closed, and intravenous 1 g at 3 and 6 h after surgery, following the standardized perioperative protocol used at the Institution. Pharmacological thromboprophylaxis was routinely administered with enoxaparin 4,000 IU subcutaneously once daily, continued until full weight-bearing was regained or for a maximum of 35 days after surgery. Structured physiotherapy focusing on active ankle range of motion, gait training through assisted ambulation with crutches, and standardized discharge criteria based on mobility, pain control, and wound status [17–20]. According to the fast-track protocol, active ankle mobilization was planned to begin approximately 6 h after surgery. Assisted mobilization with a walker and partial weight-bearing was planned from approximately 12 h after surgery, provided that the patient was clinically stable and able to participate safely. From postoperative day 2, patients were progressively transitioned to crutches with weight-bearing as tolerated, while supervised passive and active ankle range-of-motion exercises and physiotherapy were continued. By the third postoperative day, the patient continued ambulation with crutches while maintaining the weight-bearing-as-tolerated protocol. Stair climbing was introduced at this stage. The patient was instructed to ascend and descend one step at a time, leading with the operated limb when ascending and with the non-operated limb when descending.

The predefined times of approximately 6 h for active ankle mobilization and 12 h for walker-assisted partial weight-bearing represented protocol targets rather than fixed outcome measures. The mobility endpoints were based on the actual achievement of each postoperative milestone, with time zero defined as the end of surgery. Thus, time to active ankle mobilization and time to assisted ambulation with crutches were calculated from the end of surgery to the first documented achievement of the respective milestone. Time was recorded in postoperative days, including half-day increments when applicable.

### Outcome measures

Patient demographic characteristics, such as age, sex, and comorbidities, were extracted from clinical records by trained clinicians using standardized forms. All data were anonymized and securely stored. Primary and secondary outcomes were prospectively collected. The primary outcome was the ankle function assessment, measured using the AOFAS Ankle Hindfoot Scale [21]. The AOFAS Ankle-Hindfoot Scale ranges from 0 to 100 points, with higher scores indicating better clinical function. The AOFAS Ankle-Hindfoot Scale was assessed preoperatively and at hospital discharge as an exploratory measure of early postoperative clinical status. The secondary outcomes were the early postoperative recovery (days to active ankle mobilization and days to assisted ambulation with crutches) and the length of the hospital stay in days. For exploratory interpretation of the change in AOFAS Ankle-Hindfoot Scale scores, previously reported MCID estimates were considered [22, 23]. Because no universally accepted MCID has been established specifically for patients undergoing total ankle replacement, values reported in other foot and ankle populations were used only as contextual references rather than as a predefined threshold for clinical success.

Early postoperative wound status was assessed during the first 5 postoperative days using the ASEPSIS scoring system [24]. For the present analysis, the ASEPSIS score recorded at discharge was used as the reported early wound-healing outcome. The ASEPSIS score quantifies wound healing based on the presence and severity of serous discharge, erythema, purulent exudate, separation of wound edges, and additional clinical interventions required, such as antibiotic therapy, drainage procedures, or prolonged hospitalization. Each component is assigned a weighted score, allowing for a standardized assessment of wound integrity over time. According to the established ASEPSIS thresholds, scores < 10 were considered indicative of satisfactory wound healing, whereas higher scores indicated increasing wound-related severity. Late wound complications were assessed separately during the postoperative follow-up of at least 12 months. Follow-up was performed for at least 12 months primarily for safety surveillance, including assessment of wound complications, wound dehiscence, infection, prosthesis-related adverse events, and reoperations. Standardized functional and radiological outcomes were not prospectively collected at the 12-month time point. Data were collected by a healthcare professional independent of the operating surgeon (orthopaedic surgeon, physiatrist, or physiotherapist), who was not involved in the surgical procedure.

### Statistical analysis

All data were measured, collected, and reported to one decimal accuracy. Continuous variables were reported as median and interquartile range (IQR); counts for the categorical variables were recorded. The Shapiro–Wilk test was used to assess the normality of baseline AOFAS Ankle Hindfoot Scale scores. Non-normally distributed paired data were compared using the Wilcoxon signed-rank test. Median differences were reported with 95% confidence intervals (CI). A p-value < 0.05 was considered statistically significant. Exploratory associations between preoperative AOFAS Ankle Hindfoot Scale score and postoperative endpoints, including days to active ankle mobilization, days to assisted ambulation with crutches, length of hospital stay, and ASEPSIS wound score, were evaluated using Spearman rank correlation coefficients. Generalized linear models (GLMs) with a Gamma distribution and a log link were also fitted for each postoperative endpoint, with preoperative AOFAS score as the sole predictor. Given the small sample size, HC3 heteroskedasticity-consistent robust standard errors were used for the GLM estimates and corresponding 95% confidence intervals. These analyses were considered exploratory, and the associations were not interpreted as causal. Non-parametric Spearman correlation coefficients were also calculated as descriptive analyses. All analyses were performed using R 4.6.1 (R Foundation for Statistical Computing, Vienna, Austria).

## Results

The characteristics of the included patients and their functional data are summarized in Table 1. Twenty-eight patients were screened for eligibility. Five patients were excluded because they did not meet the predefined inclusion criteria, and one patient declined participation. The remaining 22 patients were enrolled and completed the study and were included in the final analysis. No additional protocol deviations or losses to follow-up occurred.There were 13 (59.0%) female and 9 (40.9%) male patients. The median age was 54.5 years (IQR, 47.5–63.0; 95% CI, 46.0–65.0), while the median BMI in kg/m² was 27.7 (26.4–29.2). Two patients (9%) were affected by diabetes mellitus, not insulin-dependent, and one (4.5%) experienced an acute myocardial infarction one year before the intervention. The primary indications for surgery were end-stage post-traumatic osteoarthritis (86.4%) and rheumatoid arthritis (13.6%). Preoperative AOFAS Ankle Hindfoot Scale score showed a median value of 29.0 (IQR, 27.0–34.0; 95% CI, 26.0–34.0). Postoperative AOFAS Ankle Hindfoot Scale scores showed a median of 68.0 (IQR, 61.0–79.0; 95% CI, 61.0–79.0). Comparing pre- and postoperative AOFAS Ankle Hindfoot Scale scores, a statistically significant median functional gain of 39 points was found (*p* < 0.001). Early postoperative recovery was achieved under the fast-track protocol: active ankle mobilization started after a median of 2 days (IQR, 2–3; 95% CI, 2–3), and assisted ambulation with crutches was attained at a median of 3 days (IQR, 2.5–3.0; 95% CI, 2.5–3.5). These values represent the actual postoperative milestones achieved by the patients and therefore differ from the earlier time points specified as targets within the fast-track protocol. The median length of hospital stay was 4.5 days (IQR, 3–5.8; 95% CI, 4–6).

Postoperative wound healing was acceptable, with a median ASEPSIS score of 4 (IQR, 4–4; 95% CI, 4–4.5). Eighteen patients had a score of 4, three patients had a score of 3, and one patient had a score of 7. All patients had a discharge ASEPSIS score < 10, corresponding to satisfactory wound healing according to the predefined threshold. No major wound complications, early infections, or prosthesis-related adverse events were observed in the short-term and during the 12-month follow-up period.

**Fig. 3:**
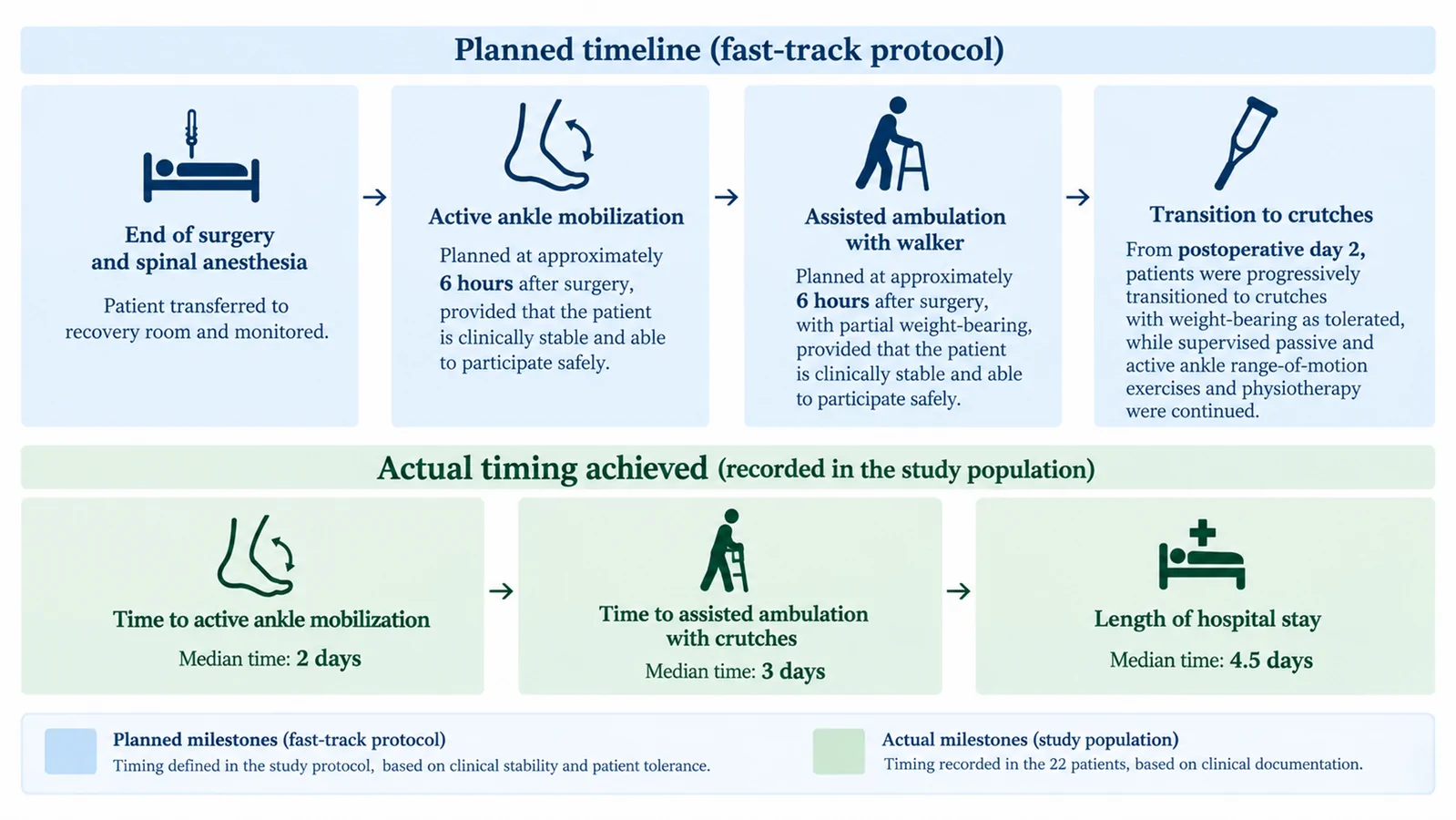
Clinical protocol of the study

**Fig. 4:**
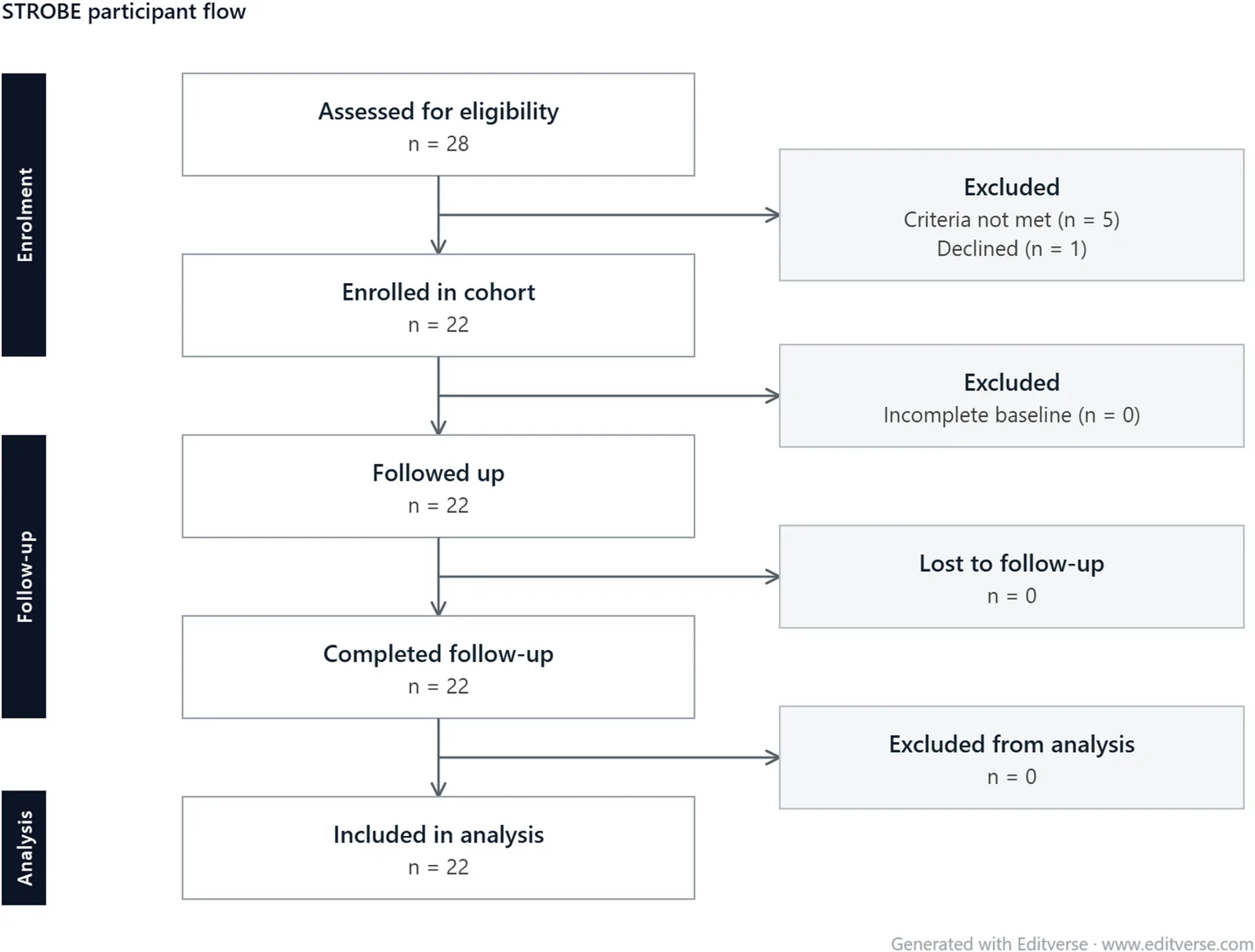
Study flow-chart

Regarding the associations between preoperative AOFAS Ankle Hindfoot Scale score and postoperative endpoints, higher preoperative AOFAS Ankle Hindfoot Scale scores were significantly associated with shorter times to active ankle mobilization (β=-0.035, 95% CI -0.058 to -0.012, *p* = 0.003), shorter time to assisted ambulation (β=-0.028, 95% CI -0.049 to -0.007, *p* = 0.010), and shorter length of hospital stay (β=-0.024, 95% CI -0.047 to -0.001, *p* = 0.042). ASEPSIS wound score showed a trend toward lower scores with higher AOFAS Ankle Hindfoot Scale (β=-0.021, 95% CI -0.043 to 0.001, *p* = 0.058). Each point increase in preoperative AOFAS Ankle Hindfoot Scale was associated with a 3.5% reduction in days to mobilization, 2.8% reduction in days to assisted ambulation, 2.4% reduction in hospital stay, and a trend toward lower ASEPSIS score (≈ 2%), although the latter did not reach statistical significance (*p* = 0.058). Spearman correlations confirmed these associations (ρ range 0.43–0.61, all *p* ≤ 0.05), except ASEPSIS (*p* = 0.058) (Table 2). Figure 3 provides a concise visual overview of the overall clinical pathway, while Fig. 4 depicts the study flow chart.

**Table 1:** Demographic characteristics of included subjects

	Age	Sex	BMI	Smoking status	ASA status	Comorbidity	Indications for surgery	Pre-operative AOFAS	Post-operative AOFAS	Days to active ankle mobilization	Days to assisted ambulation with crutches	Length of the hospital stay (days)	ASEPSIS wound score
1	37	M	24.8	Never	II		Post-traumatic arthritis	28	62	1	2.5	5	3
2	47	M	27.1	Former	II		Post-traumatic arthritis	30	79	1	2.5	5	3
3	39	M	25.6	Never	I		Post-traumatic arthritis	29	63	1	2.5	4	3
4	41	M	28.3	Never	II		Post-traumatic arthritis	29	73	2	3	3	4
5	70	F	26.7	Never	II		Post-traumatic arthritis	16	58	2	3	7	4
6	44	F	24.1	Never	I		Post-traumatic arthritis	41	73	2	1	3	4
7	46	F	29.2	Former	II		Post-traumatic arthritis	37	65	3	3	4	4
8	48	M	27.8	Current	II		Post-traumatic arthritis	32	68	2	3	5	4
9	49	F	25.9	Never	II		Post-traumatic arthritis	29	62	2	2	5	4
10	51	F	30.1	Former	II		Rheumatoid arthritis	34	71	2	3	4	4
11	52	M	28.5	Former	III	Myocardial infarction	Post-traumatic arthritis	26	58	2	2	7	4
12	54	F	26.3	Never	II		Post-traumatic arthritis	41	95	2	3	3	4
13	55	F	31.0	Never	II		Post-traumatic arthritis	30	79	2	3	3	4
14	57	M	27.4	Current	II		Rheumatoid arthritis	29	61	2	3	6	4
15	59	M	29.0	Former	II		Post-traumatic arthritis	32	62	2	3	3	4
16	60	F	24.9	Never	II		Post-traumatic arthritis	28	58	2	3	6	4
17	62	F	28.2	Never	II		Post-traumatic arthritis	34	83	2	3	3	4
18	63	F	30.4	Former	II		Rheumatoid arthritis	26	76	3	3	4	4
19	65	M	27.6	Former	III	Diabetes mellitus	Post-traumatic arthritis	41	90	2	3	3	4
20	66	F	26.8	Never	II		Post-traumatic arthritis	27	68	3	3.5	6	4
21	68	F	29.5	Never	II		Post-traumatic arthritis	28	87	3	3	5	4
22	70	F	32.1	Former	III	Diabetes mellitus	Post-traumatic arthritis	12	61	3	3.5	7	7
Overall	54.5 (47.3–62.8)	9 M/13F	27.7 (26.4–29.2)					29.0 (28–33.5)	68 (62.0–78.3)	2 (2–2)	3 (2.6–3.0)	4.5 (3–5.8)	4 (4–4)

Data are presented as medians (interquartile ranges), unless otherwise indicated

*M* male, *F* female, *AOFAS* AOFAS Ankle Hindfoot Scale

**Table 2:** Associations between preoperative AOFAS Ankle-Hindfoot Scale score and postoperative endpoints

Outcome	Spearman ρ	*p*-value	Gamma GLM β	95% CI	Robust *p*-value
Days to active ankle mobilization	− 0.24	0.285	− 0.007	− 0.019 to 0.005	0.238
Days to assisted ambulation with crutches	− 0.17	0.459	− 0.009	− 0.024 to 0.006	0.231
Length of hospital stay	− 0.77	< 0.001	− 0.034	− 0.041 to − 0.026	< 0.001
ASEPSIS wound score	− 0.15	0.505	− 0.011	− 0.024 to 0.003	0.137

Spearman rank correlations were calculated as non-parametric exploratory analyses. Gamma GLMs used a log link with preoperative AOFAS score as the sole predictor. Robust HC3 standard errors were used for the GLM estimates. Negative β coefficients indicate an inverse association between preoperative AOFAS score and the outcome. No causal inference should be drawn from these exploratory analyses

*AOFAS* American Orthopaedic Foot and Ankle Society Ankle-Hindfoot Scale, *GLM* generalized linear model, *CI * confidence interval, *ASEPSIS*  Assessment of Surgical Wounds Evaluation Score

## Discussion

This prospective study aimed to evaluate the short-term clinical outcomes of a fast-track protocol following TAR with an anterior approach. The data obtained demonstrated a statistically significant and clinically important improvement for the AOFAS Ankle Hindfoot Scale score. Early functional recovery was observed, with active ankle mobilization achieved after a median of two days and a median hospital stay of five days. However, in the absence of a conventional-care comparator, these findings should be interpreted as descriptive and do not demonstrate a superiority of the fast-track protocol. No wound dehiscence, nor any complications was found in the short-term and up to the 12-month follow-up period. These findings suggest that implementation of a structured fast-track pathway was associated with early functional recovery and favourable short-term safety outcomes. The clinically significant improvement in ankle function observed in this study, as reflected by the median AOFAS Ankle Hindfoot Scale score increase from 29.0 preoperatively to 68.0 at discharge, underscores the potential of structured fast-track pathways to facilitate meaningful recovery even within the immediate postoperative period.

### AOFAS ankle hindfoot scale and comparison with previous literature

Our results are in line with the previous work of Usuelli et al., who demonstrated the feasibility of a fast-track approach after TAR, finding a statistically significant improvement of the AOFAS Ankle Hindfoot Scale score in the fast-track group [25]. These findings contribute to the growing evidence supporting the application of enhanced recovery after surgery (ERAS) principles in foot and ankle surgery [8, 25–28]. These trends include a pre-operative phase with patient education to explain the procedure, the recovery process, and pain management strategies. An intra-operative phase, based on the use of regional anaesthesia, fluid therapy, and a minimally invasive approach; finally, a post-operative phase focused on analgesia and early mobilization within 24–48 h. Similar trends have been reported in enhanced recovery programs for hip and knee- arthroplasty, where early mobilization and multimodal rehabilitation protocols have consistently resulted in faster restoration of joint function and improved patient satisfaction [29, 30]. Furthermore, as concluded in a large observational study of 4,500 cases, patients showed a substantial reduction in mortality, reduced length of stay, and reduced transfusion requirements after the introduction of a multimodal enhanced recovery protocol [31].

### Early mobilization

In our series, patients achieved active ankle mobilization within a median of two days and assisted ambulation within three days, demonstrating that early rehabilitation is feasible and well-tolerated after TAR. These results align with earlier reports by Wang and Li [32] and Bretherton et al. [33], who emphasized that early functional loading and movement can be safely implemented following ankle replacement, provided that patient selection, surgical technique, and wound management are optimized. The benefits of early mobilization extend beyond joint-specific outcomes. Previous research in lower limb arthroplasty has shown that starting movement within the first postoperative days can reduce stiffness, improve synovial fluid circulation, and decrease muscle atrophy [34]. Moreover, early ambulation has been associated with a lower incidence of thromboembolic events and pulmonary complications, improving overall postoperative safety [35]. Within this context, the results of our study reinforce the feasibility and safety of early mobilization after TAR when performed under a structured and supervised fast-track protocol.

### Length of hospital stay

Further relevant finding was the median hospital length of stay of five days in our cohort. Although this duration appears favourable when considered alongside previously reported hospital stays after TAR, the absence of a control group prevents us from concluding that the fast-track protocol resulted in a superiority. This reduction is clinically relevant, particularly in healthcare systems increasingly focused on optimizing bed occupancy and cost efficiency. In hip and knee arthroplasty, the implementation of ERAS protocols has been shown to decrease hospital length of stays by 30–50% maintaining safety and improving patient satisfaction and without increasing readmission or complication rates [36–38]. Our findings suggest that similar benefits can be achieved in ankle replacement, which remains a less commonly performed but technically demanding procedure with traditionally longer recovery times. The benefit of early mobilization derives from physiological mechanisms, including fluid and blood circulation, decreased joint stiffness, and prevention of postoperative muscle atrophy. Multimodal pain management and the use of dexamethasone in the perioperative period may further reduce inflammation, facilitate movement and enhance patient comfort.

### Wound healing and safety

Our results also suggest that early and controlled mobilization in a fast-track protocol does not compromise wound integrity. In fact, the median ASEPSIS score of 4 indicate satisfactory healing with only one minor wound complication managed conservatively. Importantly, no major wound complications or prosthesis-related adverse events occurred. These results are encouraging, given that wound complications remain a major concern in TAR, often attributed to limited soft-tissue coverage and the anterior surgical approach [39–41]. This supports the growing perception that well-designed enhanced postoperative protocols can maintain surgical safety while accelerating recovery.

### Economic and organizational implications

Beyond clinical outcomes, adopting a fast-track approach carries potential economic and organizational advantages. A fast-track approach may have potential economic and organizational advantages, particularly if it can safely facilitate earlier discharge [11]. However, because our study did not include a comparator group or a formal cost-effectiveness analysis, no conclusions regarding cost reduction can be drawn from the present data. The median hospital stay of five days observed in our cohort provides only a preliminary descriptive finding that may warrant further investigation in comparative cost-effectiveness studies, as previously suggested [37, 38]. In addition, by enabling earlier discharge and quicker return to self-care, such protocols may indirectly improve patient satisfaction and reduce the socioeconomic burden associated with postoperative immobility. The successful implementation of a fast-track TAR protocol depends on several key components, including multidisciplinary coordination, patient education, optimized pain management, and early physiotherapy involvement. Effective preoperative counselling is critical to ensure patient adherence and motivation throughout the accelerated recovery process [42–44]. Moreover, multimodal analgesia—combining regional anaesthesia and anti-inflammatory agents—has been shown to improve pain control while minimizing opioid consumption and associated side effects [45]. Integrating these principles within the perioperative management of TAR can help achieve faster mobilization while maintaining patient comfort and safety.

### Limitations

While the findings of this study are promising, several limitations must be acknowledged. The relatively small sample size and absence of a control group limit the generalizability of our results and preclude direct comparison with conventional postoperative care. Furthermore, because no conventional-care control group was included, the observed improvements cannot be attributed exclusively to the fast-track protocol, as postoperative recovery may also have been influenced by the surgical procedure, patient characteristics, perioperative management, and the natural course of recovery. We excluded cases with neurological disorders of the lower limbs, and clinical scores were not considered to evaluate bone quality or mental status [46, 47]. These conditions may be present in other patients and may affect their postoperative outcomes; moreover, these factors could influence the decision for treatment and rehabilitation in addition to having already been associated with impaired functional recovery after various orthopedic procedures [48, 49]. Additionally, patient-reported outcomes were evaluated only at discharge, and longer-term follow-up data regarding sustained functional improvement and potential late complications are lacking. Particularly, the AOFAS Ankle-Hindfoot Scale was assessed at hospital discharge, corresponding to a median of five days after surgery. This early assessment should be interpreted cautiously, as the recall period may overlap the immediate perioperative phase and some functional activities included in the scale may not yet be applicable at this stage following total ankle replacement. Therefore, the discharge AOFAS score should be considered an exploratory measure of early postoperative status rather than a definitive assessment of functional recovery. Moreover, some functional items of the scale may not yet be fully applicable at this stage. Although all patients underwent at least 12 months of clinical follow-up for safety surveillance and postoperative complications, standardized long-term functional and radiological outcomes were not prospectively collected.

### Future perspectives

Previous studies have indicated that early postoperative improvements do not always predict long-term outcomes, emphasizing the need for extended observation [50, 51]. Future research should therefore focus on multicentre randomized controlled trials comparing fast-track and standard rehabilitation pathways, incorporating both clinical and cost-effectiveness endpoints. Moreover, the inclusion of individualized rehabilitation strategies, such as adaptive physiotherapy programs or digital tele-rehabilitation platforms, could further enhance recovery and patient engagement [52]. Tele-rehabilitation, in particular, has shown promising results in total knee and hip arthroplasty, offering continuous patient monitoring, improved adherence, and reduced travel burden [53–55]. The integration of similar technologies in TAR recovery protocols may further support early mobilization and optimize long-term outcomes. In summary, this study provides data suggesting that a structured fast-track protocol after TAR can facilitate early functional recovery without compromising safety or wound healing. This approach can be more efficient, patient-centred, and economically sustainable. Potential drawbacks of our study are the limited number of patients and the short-term follow-up. Larger and longer follow-up studies are therefore important.

## Conclusion

The data from this small prospective single-arm study suggest that a structured fast-track protocol following total ankle replacement is feasible and can be implemented with early mobilization and favourable short-term safety outcomes. The median hospital stay of five days represents a descriptive finding and further comparative studies with larger cohorts and longer follow-up are required to determine whether fast-track pathways can improve clinical outcomes and reduce hospitalization and healthcare costs compared with standard postoperative management. Integration of a fast-track protocol into routine clinical practice for ankle arthroplasty could offer a pathway for optimizing both patient outcomes and healthcare efficiency.

## Supplementary Information

Below is the link to the electronic supplementary material.

Supplementary Material 1

## Data Availability

No datasets were generated or analysed during the current study.
