# Supplementary material for: Clinical outcomes and feasibility of a fast-track protocol after total ankle replacement: a prospective study
Source: Arch Orthop Trauma Surg. 2026 Oct 20;146(1):372. doi: 10.1007/s00402-026-06532-9 (PMC13683537; doi:10.1007/s00402-026-06532-9)
Supplement: Supplementary file 1 [file 402_2026_6532_MOESM1_ESM.docx]

| **Timing** | **Intervention / Target** | **Details** | **Responsible staff** |
| --- | --- | --- | --- |
| **Perioperative period** | Multimodal analgesia | Multimodal pain management according to the institutional protocol | Anesthesiologist / medical staff |
| **Perioperative period** | Bleeding control | Tranexamic acid administration according to the institutional protocol | Anesthesiologist / medical staff |
| **Perioperative period** | Anti-inflammatory management | Dexamethasone administration according to the institutional protocol | Anesthesiologist / medical staff |
|  | | | |
| **Within 3 h after surgery** | Early mobilization | Initial mobilization and clinical assessment | Medical + rehabilitation staff |
| **Within 3 h after surgery** | Cryotherapy | Application of cryotherapy | Rehabilitation/nursing staff |
|  | | | |
| **Postoperative Day 1** | Active ankle ROM | Active range-of-motion exercises | Physiotherapist |
| **Postoperative Day 1** | Mobilization | Progressive mobilization according to clinical tolerance | Physiotherapist |
|  | | | |
| **6 h after surgery / Day 1** | Ambulation | Walking with a walker and partial weight-bearing | Physiotherapist |
|  | | | |
| **Postoperative Day 2** | Ambulation progression | Walking with crutches and weight-bearing as tolerated (WBAT) | Physiotherapist |
| **Postoperative Day 3** | Functional progression | Stair training | Physiotherapist |
|  | | | |
| **Throughout hospitalization** | Pain control | Regular assessment and multimodal analgesia | Medical + nursing staff |
| **Throughout hospitalization** | Wound monitoring | Clinical assessment of wound healing and postoperative complications | Medical + nursing staff |
|  | | | |
| **Before discharge** | Discharge assessment | Clinical stability, adequate pain control, functional mobility, and ability to perform the required activities safely | Medical + rehabilitation staff |
